# Novel evolved *Yarrowia lipolytica* strains for enhanced growth and lipid content under high concentrations of crude glycerol

**DOI:** 10.1186/s12934-023-02072-8

**Published:** 2023-03-31

**Authors:** Asimina Tsirigka, Eleni Theodosiou, Sotiris I. Patsios, Antiopi Tsoureki, Aggeliki Andreadelli, Elisavet Papa, Amalia Aggeli, Anastasios J. Karabelas, Antonios M. Makris

**Affiliations:** 1 Laboratory of Natural Resources and Renewable Energies, Chemical Process and Energy Resources Institute, Centre for Research and Technology - Hellas, Thermi, Thessaloniki, Greece; 2 Department of Chemical Engineering, Aristotle University of Thessaloniki, Thessaloniki, Greece; 3 Institute of Applied Biosciences, Centre for Research and Technology - Hellas, Thermi, Thessaloniki, Greece; 4 Department of Computer Science and Biomedical Informatics, University of Thessaly, Lamia, Greece

**Keywords:** *Yarrowia lipolytica*, Crude glycerol valorization, Adaptive Laboratory Evolution, EMS mutagenesis, Differential gene expression

## Abstract

**Background:**

*Yarrowia lipolytica* is a well-studied oleaginous yeast known for its ability to accumulate and store intracellular lipids, while growing on diverse, non-conventional substrates. Amongst them, crude glycerol, a low-cost by-product of the biodiesel industry, appears to be an interesting option for scaling up a sustainable single-cell oil production process. Adaptive laboratory evolution (ALE) is a powerful tool to force metabolic adaptations endowing tolerance to stressful environmental conditions, generating superior phenotypes with industrial relevance.

**Results:**

*Y. lipolytica* MUCL 28849 underwent ALE in a synthetic medium with increasing concentration of pure or crude glycerol as a stressing factor (9–20% v/v) for 520 generations. In one case of pure glycerol, chemical mutagenesis with ethyl methanesulfonate (EMS) was applied prior to ALE. Growth profile, biomass production and lipid content of 660 evolved strains (EVS), revealed 5 superior isolates; exhibiting from 1.9 to 3.6-fold increase of dry biomass and from 1.1 to 1.6-fold increase of lipid concentration compared to the parental strain, when grown in 15% v/v crude glycerol. NGS for differential gene expression analysis, showed induced expression in all EVS affecting nucleosomal structure and regulation of transcription. As strains differentiated, further changes accumulated in membrane transport and protein transport processes. Genes involved in glycerol catabolism and triacylglycerol biosynthesis were overexpressed in two EVS. Mismatches and gaps in the expressed sequences identified altered splicing and mutations in the EVS, with most of them, affecting different components of septin ring formation in the budding process. The selected YLE155 EVS, used for scale-up cultivation in a 3L benchtop bioreactor with 20% v/v crude glycerol, achieved extended exponential phase, twofold increase of dry biomass and lipid yields at 48 h, while citric acid secretion and glycerol consumption rates were 40% and 50% lower, respectively, compared to the parental strain, after 24 h of cultivation.

**Conclusion:**

ALE and EMS-ALE under increasing concentrations of pure or crude glycerol generated novel *Y. lipolytica* strains with enhanced biomass and lipid content. Differential gene expression analysis and scale-up of YLE155, illustrated the potential of the evolved strains to serve as suitable “chassis” for rational engineering approaches towards both increased lipid accumulation, and production of high-added value compounds, through efficient utilization of crude glycerol.

**Supplementary Information:**

The online version contains supplementary material available at 10.1186/s12934-023-02072-8.

## Background

*Yarrowia lipolytica* is a well-studied oleaginous yeast, known for its great potential to accumulate and store intracellular lipids that may serve as raw material for 2nd generation biodiesel production [1–4]. Apart from the productive lipogenic pathways, *Y. lipolytica* is also known for the ability to utilize versatile carbon sources, such as industrial organic wastes [5, 6], including crude glycerol [2, 7, 8] and waste cooking oils [9], volatile fatty acids [10], olive oil [11] and industrial fats, such as stearin [11, 12]. Driven by biobased economy challenges, various biotech research groups design and develop ways to valorize these renewable waste materials into lipids, with a special emphasis on crude glycerol since it is a low-cost by-product of biodiesel industry and can be used without any special pretreatment [2, 13–16]. At the same time, crude glycerol does not contribute to the energy-food competition, as vegetable oils do, minimizing related ethical concerns. Considering that in 2021, the consumption of biodiesel and jet biofuel reached 7 billion liters, which is expected to grow significantly over 2021–2026, efficient carbon conversion into high added-products is critical [17].

Several research groups have described the involved lipogenic pathways and proposed strain and process engineering strategies to increase the lipid content of the yeast [2, 3, 8, 10, 18–20]. Strain optimization was further accelerated by the development of novel molecular biology tools based on the fully sequenced whole genomes of various strains [5]. Most of the engineering approaches promoted triacylglycerol (TAG) accumulation by over-expressing genes related to Kennedy pathway or by eliminating *β*-oxidation pathway [21]. For example, the simultaneous over-expression of stearoyl-CoA desaturase (*SCD*), diacylglyceride acyl-transferase (*DGA1*) and acetyl-CoA carboxylase (*ACC1*) genes, resulted in lipid titers up to 35 g/L and 0.23 g/g TAG production yield when *Y. lipolytica* was grown in high crude glycerol concentration (150 g/L) [22]. Regarding process optimization for efficient glycerol utilization, from flask to bench-bioreactor scale, the main focus targets the fine-tuning of process parameters to increase lipid and biomass production. It is known, that glycerol is the preferred carbon source for *Y. lipolytica* and when it co-exists with either glucose or xylose in the cultivation medium, it is consumed first [23]. A broad range of glycerol concentration has been tested in batch mode fermentations as a sole carbon source; from 10 to 170 g/L, whereas kinetics differ depending on the fermentation conditions [2, 14, 19, 24–26]. Research on batch cultivation of *Y. lipolytica* under higher concentrations of crude glycerol, as sole carbon source, is scarce due to the imposed osmotic stress which consequently causes severe inhibition on yeast growth performance [26]. Dobrowolski et al. studied the scale-up from flask to 2 L bioreactor fermentations supplementing with 150 g/L (12% v/v) crude glycerol, while testing two different carbon to nitrogen ratios i.e., 75 and 100 [24]. The main conclusion was that lipogenesis was enhanced when C/N = 100 was employed, which resulted in biomass and lipid yields equal to 0.166 (g_biomass_/g_glycerol_) and 0.031 (g_lipids_/g_glycerol_), respectively, after 96 h of cultivation. Similarly using crude glycerol as carbon source, Sarris et al. evaluated the performance of a *Y. lipolytica* strain in submerged shake-flask nitrogen-limited fermentations, supplemented with 170 g/L (~ 14% v/v) of crude glycerol, mixed with olive mill wastewaters and reported a biomass yield of 0.09 (g_biomass_/g_glycerol_) after 408 h of cultivation [25].

In addition to rational metabolic engineering and fine tuning of medium and cultivation conditions, adaptive laboratory evolution (ALE) [27] can be a valuable strain engineering tool. ALE is a technique where a microorganism is cultured over multiple generations under a specific selection pressure (e.g., nutrient composition, osmotic stress, pH), and its fitness improves as beneficial mutations in the population are fixed over time, while metabolism rewires to support the enhanced phenotype. Stress imposed by altering either the type or the concentration of the carbon source is a very common ALE practice to engineer superior producer strains with industrial relevance [28–32]. Various studies applied ALE to engineer *Y. lipolytica*; however, those focusing on lipid production from glycerol are scarce. Daskalaki et al. [33], reported a 30% increase of the lipid content of *Y. lipolytica* after 77 generations under stress imposed through nitrogen and magnesium starvation while using glucose as carbon source. The obtained population showed different responses upon selenium supplementation regarding the lipid composition [34]. Liu et al. applied ethyl methanesulfonate (EMS) mutagenesis on a *Y. lipolytica* strain (pre-engineered targeting lipogenesis enrichment) and conducted floating cell selection in 5 transfers for further lipogenesis enrichment, arguing that floating cells contain higher percentages of intracellular lipids, thus promoting fatty acid formation metabolic pathways [35].

In this study, we employed ALE to develop novel tolerant strains of *Y. lipolytica*, capable of growing in synthetic media containing high levels of pure and crude glycerol (~ 250 g/L, 20% v/v) as sole carbon source, while retaining or even enhancing biomass and lipid formation. Superior strains, displaying higher biomass and lipid content compared to the parental strain were selected for further characterization and scale-up cultivations with crude glycerol using a 3L benchtop bioreactor. Next generation sequencing and transcriptome analysis was used to unravel adaptation mechanisms of *Y. lipolytica* activated in media with high glycerol content and to guide future rational strain engineering.

## Results

### Adaptive laboratory evolution of *Y. lipolytica* towards tolerance to high concentrations of glycerol

**Fig. 1:**
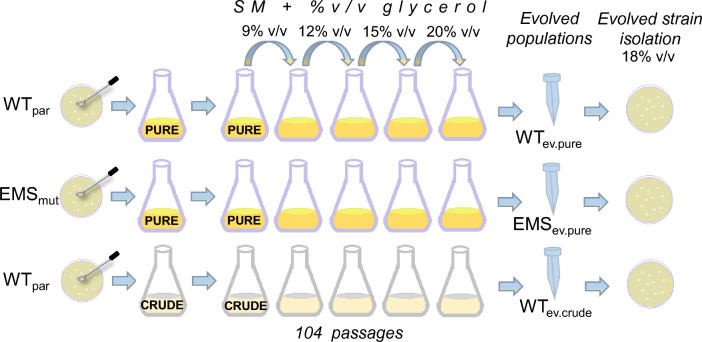
Schematic representation of ALE of *Yarrowia lipolytica*. Three ALE strategies were designed to increase biomass and lipid formation under high pure and crude glycerol concentrations. After 104 passages, evolved populations were cryo-preserved and evolved single colonies were isolated. WT_par_, *Y. lipolytica* 28849 parental strain; EMS_mut_, after random chemical mutagenesis with ethyl methanesulfonate (EMS) exposure

**Fig. 2:**
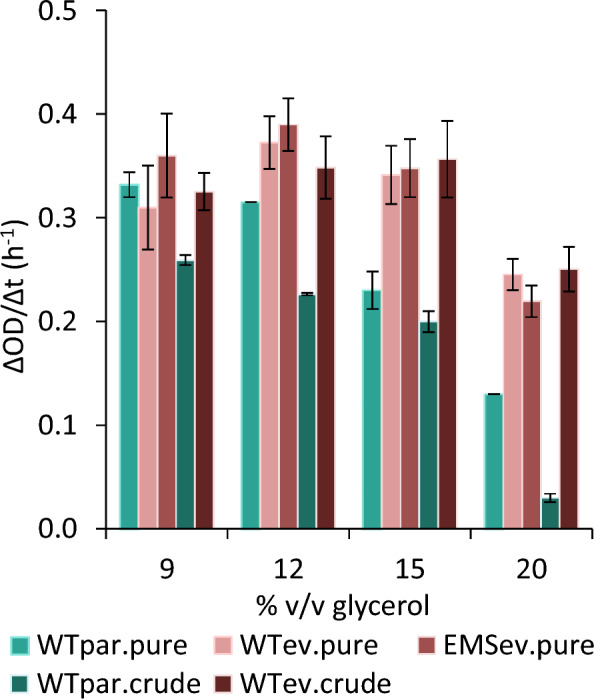
Impact of the increasing concentration (% v/v) of pure and crude glycerol on the ΔOD/Δt during ALE experiment. Monitoring of the parental strain (WT_par.pure_) grown in increasing pure glycerol concentration (light green) in comparison to the ΔOD/Δt of WT_ev.pure_ and EMS_ev.pure_ evolved populations. Monitoring of the parental strain (WT_par.crude_) grown in increasing crude glycerol concentration (dark green) in comparison to the ΔOD/Δt of WT_ev.crude_ evolved population. All other medium components were unchanged during the 104 serial transfers

For the ALE, both parental and EMS mutagenized populations underwent 104 serial subcultures as described in the Materials and Μethods section. A graphical description is depicted (Fig. 1), presenting the three ALE strategies (increased pure glycerol, increased pure glycerol after EMS mutagenesis, and increased crude glycerol). In each passage (24 h), the population growth was equal to 5 doublings, resulting in approximately 520 generations. The physiology of each evolved population was monitored after 24 h of cultivation and the average ΔOD/Δt rate was compared to that of the parental (WT_par_). The ΔOD/Δt of all three populations drastically improved over time when using pure or crude glycerol, which can be attributed to a shorter lag phase due to adaptation to high glycerol presence in the medium. As the amount of glycerol increased, the difference between the (ΔOD/Δt)_WTpar_ and the (ΔOD/Δt) of the evolved populations increased with the highest difference found at the highest glycerol concentration (20% v/v) in all ALE experiments (Fig. 2). Cell growth impairment of the parental strain was evident and proportional to the increase of glycerol concentration. This phenomenon occurred when either pure or crude was used and can be attributed to osmotic stress and the possibly toxic substances present in crude glycerol [36, 37]. For that reason, osmolality of the culture media was determined for each pure and crude glycerol concentration (9–20% v/v) and varied from 1,682 to 3,793 mOsmol/kg and from 2,241 to 5,619 mOsmol/kg, respectively (Additional file 1: Table S1). The increased osmolality of the crude glycerol synthetic medium, can be associated to the impurities of the carbon source, since the purity of crude glycerol was approximately 90–92%, which was taken into account to reach the respective carbon concentrations (9–20% v/v). Specifically, when 20% v/v pure or crude glycerol was used, the (ΔOD/Δt)_WTpar_ was estimated to be 0.13 h^−1^ and 0.03 h^−1^, respectively, whereas for the adapted populations i.e., WT_ev.pure_, WT_ev.crude_ and EMS_ev.pure_, ΔOD/Δt rate reached 0.25 ± 0.02 h^−1^, 0.25 ± 0.02 h^−1^ and 0.22 ± 0.02 h^−1^, respectively. ALE was completed once the phenotype of each population was evidently improved and stable in terms of ΔOD/Δt rate (ca. after 25 generations). It is notable, that by the end of the ALE experiment, the populations that evolved at elevated concentration of crude glycerol reached a similar ΔOD/Δt rate to that of pure glycerol; thus, indicating adaptation mechanisms endowing cells with improved tolerance upon the inhibitory compounds present in crude glycerol.

### Selection of superior evolved *Y. lipolytica* strains regarding biomass formation and lipid content

**Fig. 3:**
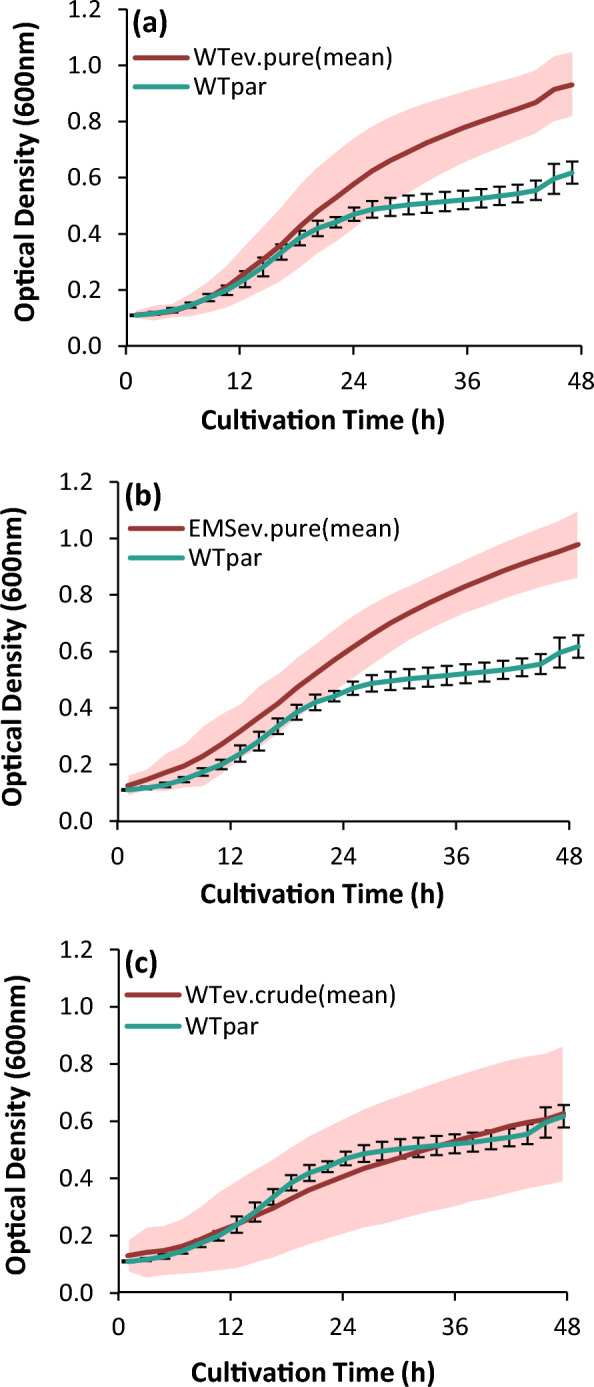
Growth profiles of 450 isolated evolved stains cultivated in 0.2 mL synthetic medium containing 15% v/v pure glycerol. The average growth curves of **a** WT_ev.pure_, n = 297; **b** EMS_ev.pure_, n = 100; **c** WT_ev.crude_, n = 53 (n, number of individual strains) are shown in red and their standard deviation in pink shading. Error bars indicate standard deviation for the parental strain WT_par_. OD_600nm_ was monitored using a microtiter plate reader. Number of technical replicates (N = 3)

In order to identify and isolate superior strains adapted to high glycerol concentrations, evolved populations were grown on synthetic medium-agar plates containing 18% v/v glycerol concentration (pure or crude according to the carbon source applied during ALE) and then single colonies were transferred to 96-well plates, resulting in 660 individual strains. From these, 450 strains were randomly selected for further investigation (i.e., WT_ev.pure_, n = 297; EMS_ev.pure_, n = 100; WT_ev.crude_, n = 53). Growth was monitored over a 48-h cultivation in 0.2 mL synthetic medium containing 15% v/v pure glycerol (Fig. 3). The screening revealed a significant heterogeneity among the isolated evolved strains, as well as the inability of the crude glycerol-adapted strains to retain this trait when cultivated in pure glycerol. As seen in Fig. 3C, despite the higher final ΔOD/Δt rate of the WT_ev.crude_ population, compared to the parental when growing in crude glycerol, the average growth profile of the 53 isolated strains (originating from the same evolved population) resembled the one of the parental when pure glycerol was used. This can be attributed to the lower purity of crude glycerol (i.e., 90—92% w/w) compared to that of the pure glycerol (≥ 99.5% w/w). Hence, the available carbon source when pure glycerol was used, was slightly higher concerning carbon concentration. It is also of great importance, that the tolerance of WT_ev.crude_ population was obtained under both increased glycerol concentration, and under increased concentration of the toxic compounds contained in the crude glycerol. Therefore, when pure glycerol was used, the stressing factor of the toxic impurities was withdrawn, whereas glycerol concentration was slightly higher than crude glycerol with the same titer (i.e., 15% v/v).

After 48 h of cultivation and under 15% v/v pure glycerol, the increased biomass concentration of the evolved strains, deriving from WT_ev.pure_ and EMS_ev.pure_ populations, to that of the WT_par_, varied from 32 to 70% and from 39 to 78%, respectively. At the same time point, the more tolerant strains, derived from the WT_ev.crude_ population, attained up to 40% higher biomass concentration, compared to the WT_par_. From those, 20 with enhanced growth performance were selected for further study. In total, 90 strains (WT_ev.pure_, n = 58; EMS_ev.pure_, n = 12; WT_ev.crude_, n = 20), out of 450, were selected to further determine lipid content using flow cytometry. Selection was based on the biomass concentration to that of the parental ratio; more specifically, strains from the WT_ev.pure_, EMS_ev.pure_, and WT_ev.crude_ exhibiting higher ratios than 60%, 70% and 20%, respectively, compared to the parental strain.

**Fig. 4:**
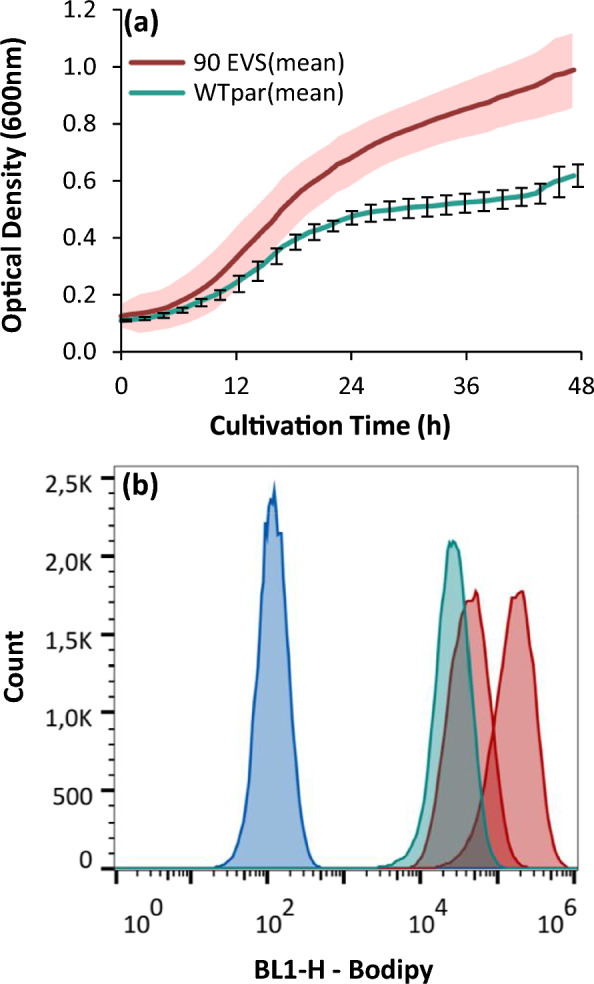
**a** Growth profiles of 90 isolated evolved strains (EVS) cultivated in 0.2 mL synthetic medium containing 15% v/v pure glycerol. The average growth curve of the EVS (WT_ev.pure_, n = 58; EMS_ev.pure_, n = 12; WT_ev.crude_, n = 20) is depicted in red while the green curve stands for the parental strain (WT_par_). Standard deviation is indicated with error bars for the WT_par_ whereas for the 90 EVS with pink shading. OD_600nm_ was monitored using a microtiter plate reader. Number of replicates (n = 3). **b** Lipid fluorescence and distribution per cell after 48 h of cultivation, using flow cytometry and bodipy dye (493/503 nm). Minimum and maximum fluorescence distribution curves of 90 EVS are shown in two red curves, WT_par_ in green, while unstained WT_par_ cells were used as control (blue)

Monitoring the growth profiles of the 90 selected evolved strains confirmed the enhanced biomass formation (Fig. 4a). One histogram per evolved strain, was obtained using flow cytometry, illustrating the distribution of the intracellular lipid fluorescent intensity of 50,000 cells. For illustrative purposes, Fig. 4b shows the two extreme fluorescence distribution curves of lipids among the 90 evolved strains in comparison with the parental. Based on lipid content, 11 strains were selected (WT_ev.pure_, n = 7; EMS_ev.pure_, n = 2; WT_ev.crude_, n = 2) which were subsequently studied in flask cultivations (25 mL, 15% v/v pure glycerol-synthetic medium).

Scaling up from 0.2 mL to 25 mL, showed that biomass productivity (P_DB_) was significantly increased after both 24 and 48 h of cultivation (Table 1) in most evolved strains (EVS). One-way ANOVA and post-hoc tests were performed to evaluate statistically significant differences between the means of each EVS and the WT_par_. Strains with improved dry biomass (DB) and lipid (L) productivity, exhibited decreased lipid yield (Y_L/DB_) compared to the WT_par_, while they reached up to 0.059 g/L/h and 0.006 g/L/h, respectively, at 48 h of cultivation. The different trend between lipid yield and lipid productivity can be attributed to the fact that lipids are intracellularly formed, thus, when DB concentration is increased, a higher total lipid concentration is achieved.

**Table 1:** Summary of dry biomass (DB) and lipid production (L) metrics of 11 selected evolved strains grown in 25 mL synthetic medium containing 15% v/v pure glycerol for 24 and 48 h. Number of biological replicates (N = 3). (Standard Deviation < 15%)

Cultivation Time (h)	24			48		
Strain No	Y_L/DB_(g/g)	P_DB_ (g/L/h)	P_L_ (g/L/h)	Y_L/DB_(g/g)	P_DB_ (g/L/h)	P_L_ (g/L/h)
WT_par_	0.210	0.014	0.0026	0.160	0.036	0.0053
YLE111	0.082*	0.054*	0.0046*	0.172	0.044	0.0064*
YLE155	0.057*	0.060*	0.0035*	0.143	0.054*	0.0058
YLE184	0.086*	0.048*	0.0041	0.134	0.064*	0.0071
YLE198	0.082*	0.035*	0.0030	0.121	0.063*	0.0057*
YLE215	0.120*	0.025*	0.0031	0.126	0.059*	0.0060*
YLE230	0.130*	0.015	0.0017*	0.140	0.048*	0.0059*
YLE348	0.059*	0.050*	0.0029	0.114	0.058*	0.0055
YLE469	0.073*	0.050*	0.0036*	0.110	0.050*	0.0047*
YLE471	0.089*	0.037*	0.0033*	0.122	0.058*	0.0064
YLE523	0.170	0.017	0.0023	0.144	0.039	0.0053
YLE562	0.220	0.012	0.0027	0.156	0.045*	0.0062

^*^Within columns indicates statistically significant difference between the means of each EVS and the WT_par_, after one-way ANOVA and post hoc tests (level of significance, 0.05)

**Fig. 5:**
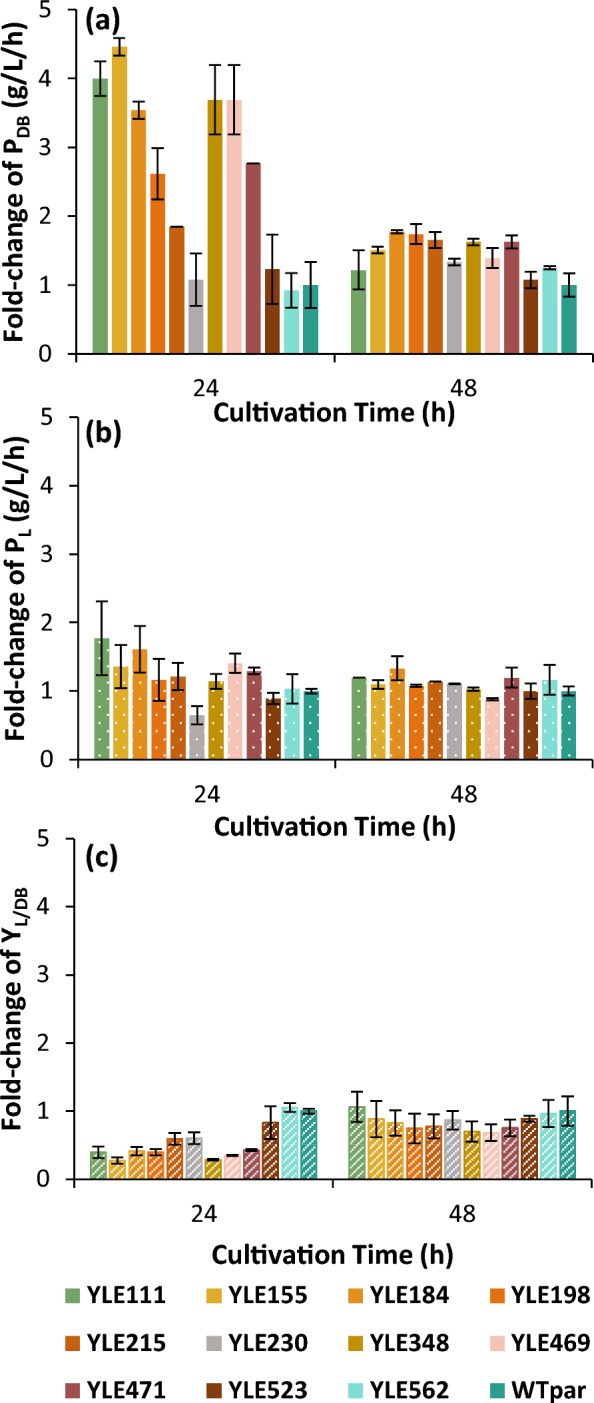
Fold-change of **a** dry biomass productivity (P_DB_), **b** lipid productivity (P_L_) and **c** lipid yield (Y_L/DB_) of 11 evolved strains (i.e., WT_ev.pure_, n = 7; WT_ev.crude_, n = 2; EMS_ev.pure_, n = 2), cultivated in 25 mL synthetic medium containing 15% v/v pure glycerol. Error bars indicate standard deviation. (Number of biological replicates N = 3)

The fold-change of dry biomass and lipid productivities of all the 11 selected strains, compared to the parental, is presented in Fig. 5. After 24 h, 5 strains, namely YLE111, YLE155, YLE184, YLE348 and YLE469, achieved a remarkable 3.5 to 4.5-fold increase of their dry biomass productivity, while 5 strains (i.e., YLE111, YLE155, YLE184, YLE469 and YLE471) showed from 1.3 to 1.8-fold increased lipid productivity. The productivity values P_DB_ and P_L_, obtained after 48 h, exhibited an increase up to 77% and 33%, respectively. The morphology of each evolved strain was similar to the parental strain apart from YLE469 (deriving from the EMS_ev.pure_ population), in which a multicellular snowflake-like phenotype was observed (Additional file 1: Figures S1, S2). This phenomenon has been observed during yeast evolution and is associated with early apoptosis [38]. It is yet to fully elucidate the mutations responsible for the shift from a yeast cell to multicellular clump morphology but it is known to be triggered in order to cope with environmental stress throughout evolution [39].

### Physiology of evolved strains when grown in crude glycerol

**Fig. 6:**
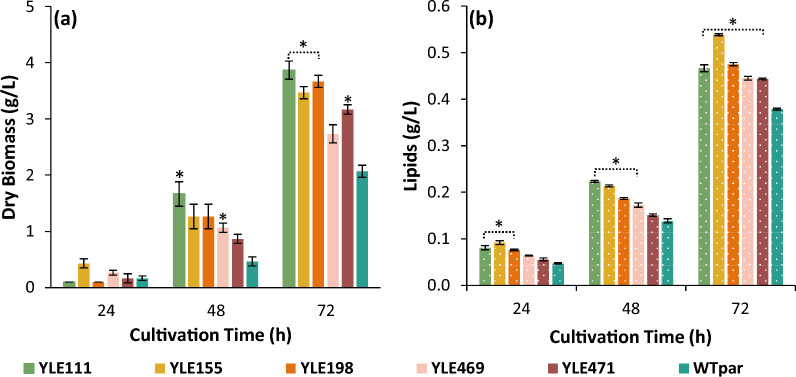
Monitoring of **a** dry biomass (g/L) and **b** lipid concentration (g/L) of 5 superior evolved strains, cultivated in 25 mL of synthetic medium, containing 15% v/v crude glycerol. Error bars indicate standard deviation. Statistically significant difference between the means of each EVS and the WT_par_, is shown with (*) (level of significance, 0.05). Number of biological replicates (N = 3)

The dry biomass and lipid results, narrowed down the number of individual selected strains to 5 (WT_ev.pure_, n = 3; EMS_ev.pure_, n = 2). Crude glycerol was used as carbon source instead of pure glycerol, to identify possible differences in fermentation kinetics. The 5 evolved strains were characterized under stressful environmental conditions in synthetic medium containing 15% v/v crude glycerol. The growth and lipid phenotype, when using biodiesel-derived crude glycerol as sole carbon source, was crucial for the final strain selection, since crude glycerol can be exploited as raw material in large-scale fermentations. Figure 6, presents the enhanced biomass and lipid formation by the evolved strains compared to the wild type, indicating that the shift to crude glycerol did not significantly affect the improved phenotypes.

All 5 strains displayed increased biomass formation and lipid production compared to the parental (Fig. 6). The fold-increase of DB and lipid concentration, varied from 1.9 to 3.6 and from 1.1 to 1.6, respectively, after 48 h of cultivation. YLE155 strain reached a 2.6-fold increase of DB in the first 24 h and YLE111 strain a 3.6-fold increase of DB after 48 h cultivation. The highest DB productivity was obtained after 48 h, with YLE111 strain reaching up to 0.035 g/L/h. Since the culture of YLE155 (in 15% v/v crude glycerol-synthetic medium) reached the maximum dry biomass and lipid concentration in 24 h (94% higher than the WT_par_), YLE155 was chosen for scale-up cultivations in a laboratory bioreactor.

### Transcriptome and differential expression analysis of the superior evolved strains

**Fig. 7:**
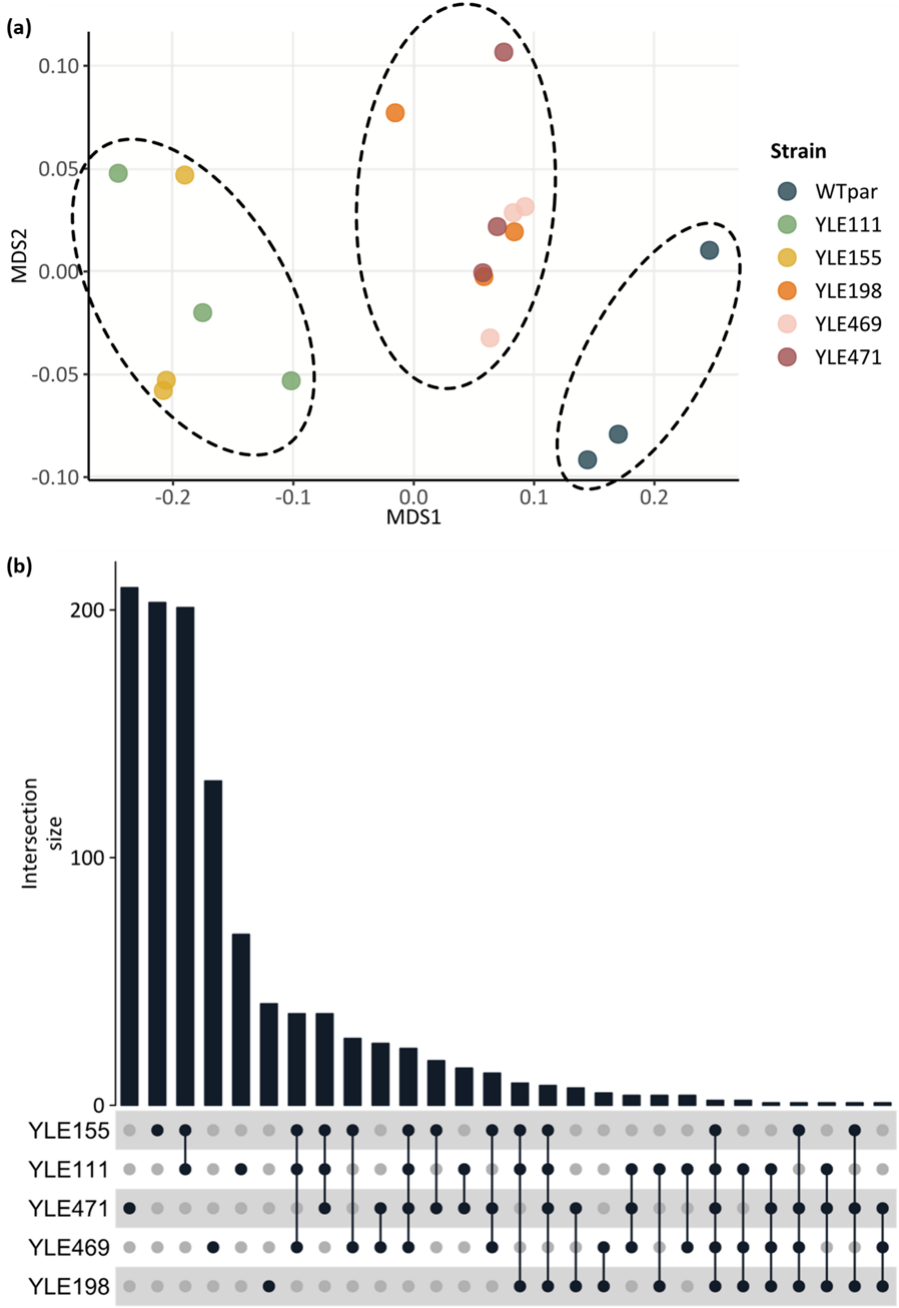
Differential gene expression analysis of the evolved *Y. lipolytica* strains. **a** Multidimensional scaling plot (MDS), for all libraries using all non-zero gene FPKMs. Biological replicates are shown in the same color. **b** UpSet plot, visualizing intersections between the sets of differentially expressed genes (DEGs) for the selected evolved strains compared to the parental (WT_par_). The height of the bar indicates the number of DEGs included in each intersection for one or more evolved strains. The dark dots under each bar show which evolved strains are considered for each intersection. Empty intersections are not shown in the plot.

Differential gene expression analysis between the WT_par_ and the superior EVS was performed by Next-Generation Sequencing (NGS). The obtained raw reads and the remaining number of reads after quality filtering are presented in the Additional file 1: table S2. In total, 8,027 genes were detected, while 6,472 genes remained after filtering out low abundance genes. Multidimensional scaling (MDS) based on the gene expression profile of the samples (Fig. 7a) showed that the evolved strains formed two separate clusters while the parental was not included in either of the two, indicating that, in terms of gene expression profile, differences were observed both between the evolved strains and the parental as well as among the various EVS. YLE111 and YLE155, derived from WT_ev.pure_ population, were characterized by similar gene expression profiles, as were YLE198 (derived from WT_ev.pure_), YLE469 and YLE471 (derived from EMS_ev.pure_).

### Differences in gene expression

The evolved strains, displaying the most differentiated gene expression profile compared to the parental_,_ were YLE111 and YLE155, with 417 and 580 differentially expressed genes (DEGs), respectively. Accordingly, evolved strains with more similar gene expression profile to that of the parental, had also fewer DEGs, i.e., YLE198; 83, YLE469; 276, YLE471; 366. The relation between the set of DEGs of each evolved strain, as shown in the UpSet plot (Fig. 7b), provides insight by firstly introducing the number of DEGs detected only in each strain individually and secondly, indicating how many of the DEGs were common among the various evolved strains. YLE111 and YLE155 had many common DEGs, whereas the same is not true for the remaining three evolved strains, despite their grouping in one cluster in the MDS plot. Two genes were differentially expressed in all evolved strains with UniProt accessions Q6C6L1 and Q6C6K2. The Gene Ontology (GO) terms in the domain of biological processes (BP), associated to Q6C6L1 and Q6C6K2, were “plasma membrane fusion involved in cytogamy” (GO:0032220) and “mitochondrial genome maintenance” (GO:0000002) processes, respectively. Alterations in expression of genes involved in cytogamy have been previously reported after imposed oxidative stress, as a stress response in *S. cerevisiae* mutants [40]. Mitochondrial genome maintenance by the means of structure and integrity of the mitochondrial genome includes replication and segregation of the mitochondrial chromosome. Based on these results, we hypothesized that *Y. lipolytica,* in order to combat the stressful environment caused by the presence of high glycerol concentration, changed the expression rates of genes participating in metabolic pathways closely related to cell fusion.

According to the glycerolipid metabolic pathway of *Y. lipolytica* in KEGG database [41], genes linked to glycerol catabolism and TAG biosynthesis, were found only in the pool of YLE111 and YLE155 DEGs. YALI0_C00209g, encoding glycerol-3-phosphate O-acyltransferase and dihydroxyacetone phosphate acyltransferase, was overexpressed in YLE111 and YLE155. YALI0_C14014g, encoding 1-acylglycerol-3-phosphate O-acyltransferase, was also overexpressed in the same evolved strains. YALI0_B14531g was also included in the overexpressed DEGs of YLE155, encoding diacylglycerol diphosphate phosphatase and phosphatidate phosphatase. YALI0_D07986g, encoding diacylglycerol O-acyltransferase 1, was not differentially expressed in any of the evolved strains.

### Biological processes affected in evolved strains

**Fig. 8:**
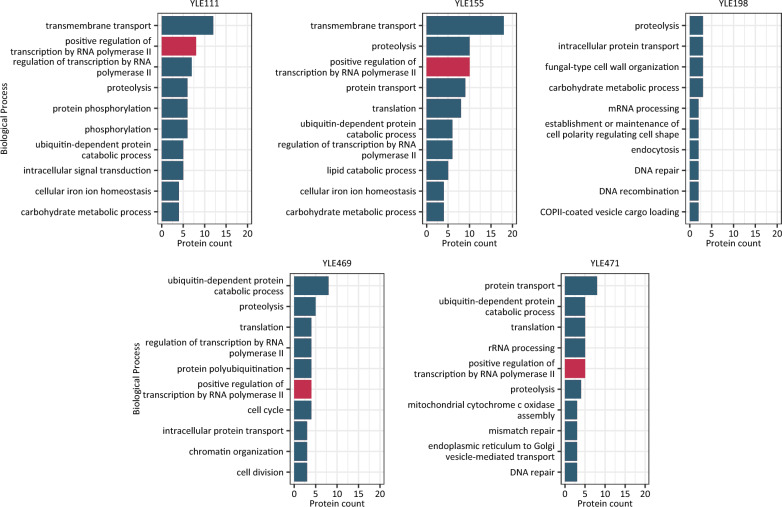
Abundance levels of the most abundant gene ontology (GO)—biological process (BP) terms associated with the differentially expressed genes (DEGs) in all 5 evolved strains (YLE111, YLE155, YLE198, YLE469 and YLE471)

To get an insight into the biological processes (BP) linked to the DEGs of each evolved strain, abundance levels of the most frequent Gene Ontology (GO:BP) terms, associated with the DEGs in the evolved strains, were determined (Fig. 8). GO annotation of all 5 strains (focused on BP) showed that most of the DEGs were related to membrane transport activation. GO terms associated with biological processes involved in lipid-related processes were spotted in YLE111 and more frequently in YLE155 strain, where five DEGs were linked to lipid catabolic processes. In more detail, these DEGs correspond to processes connected mainly to cell membrane structure and phospholipid biosynthetic process (GO:0008654), rather than synthesis and storage of TAG, apart from the gene YALI0_A10362g (found in YLE111) that is also linked to fatty acid (GO:0006631) and glycerol-3-phosphate (GO:0006072) metabolic processes, phospholipid biosynthetic process (GO:0008654) and triglyceride biosynthetic process (GO:0019432). YALI0_C24145g gene, being one of the overexpressed DEGs in YLE111 and YLE155, is also linked to lipid droplet organization (GO:0034389), lipid storage (GO:0019915) as well as phospholipid biosynthetic process (GO:0008654) GO:BP terms. The lipid-related processes, reported in GO:BP terms, corresponding to YLE198, YLE469 and YLE471 evolved strains, were limited and particularly in the strains originated from EMS-ALE. This may be attributed to the chosen time point of sampling, being during the exponential growth phase of the yeast strains. A key observation of the analysis is that initial changes in all derived evolved strains affected nucleosomal structure and regulation of transcription. These nucleosomal changes are likely an early event in adaptation to high osmotic stress that leads to an accumulation of changes as strains evolve and further differentiate. In the more differentiated evolved strains (YLE111 and YLE155), these changes globally affected membrane transport and protein transport processes. Surprisingly, the GO annotation showed this similar trend in all evolved strains, even though they originated from different ALE strategies and experiments (EMS-ALE vs ALE). This may indicate that there are important fundamental commonalities on how cells respond to high osmotic stress.

Three genes involved in transcriptional regulation were overexpressed in 4 out of 5 strains (YLE111, YLE155, YLE469 and YLE471). One of them, YALI0_D01353g, codes for a zinc finger transcription factor-like DNA binding protein that is specific to *Yarrowia* and the other two are homologues of *S. cerevisiae* genes *RAD26* and *DOT1* (YALI0_C16643g and YALI0E22715g, respectively) which affect global chromatin dynamics and histone turnover. *RAD26,* a SWI2/SNF2 family member, promotes transcriptional elongation [42]. Absence of *RAD26* results in atypical histone H2A–H2B dimer enrichment. Displacement of H2A-H2B appears to promote transcriptional activation via enhanced RNA polymerase II and transcription factor binding [43]. *DOT1* is a Histone 3 H3K79 methyltransferase and a histone chaperone, which also plays a major role in histone exchange, balancing nucleosome dynamics during transcription. Dot1p alters nucleosome accessibility at the transcribed regions of genes. Histone turnover and histone chaperone activities have been shown to be important in the context of DNA damage situations [44]. These nucleosome changes appear to be early adaptation events.

As strains further differentiated, additional chromatin regulatory genes were upregulated. These were: *HOS2 (*YALI0_C06061g), a histone deacetylase required for gene activation; *JMC2* (YALI0_B14443g), a JmjC domain family histone demethylase which promotes global demethylation of H3K4; *RAD4* (YALI0_D08756g), a DNA damage response protein [45]; and REV1 (YALI0_F09141g), a specialized Translesion Synthesis (TLS) polymerase. These genes were upregulated in YLE111, YLE155 and YLE469 strains.

In the more diverged strains, YLE111 and YLE155, HRQ1, a 3’-5’ helicase that collaborates with RAD4 in DNA repair, and a lysine specific histone demethylase 1A were also upregulated. Additionally, a second functional group of genes involved in transmembrane transport processes was also enriched. This included two Multi-Drug Resistance transporters, an ARN2 homologue of iron-chelate transporter, a BOR1 Boron efflux transporter, two vacuolar glutathione S-conjugate transporters, VBA1 a basic amino acid vacuolar permease, FLC1 a Flavin adenine dinucleotide transporter, FRE3 a ferric reductase involved in iron uptake, SMF2 homologue—a divalent metal ion transporter, and OST2 homologue—an oligopeptide transporter. These later events may also reflect additional adaptations to the synthetic growth medium.

**Fig. 9:**
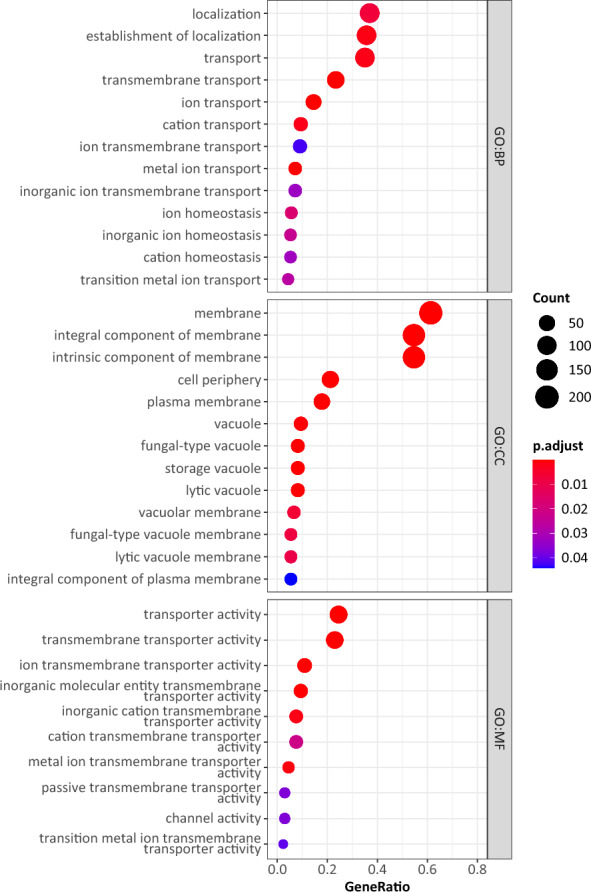
Dotplot of gene ontology (GO) enrichment analysis for YLE155 evolved *Y. lipolytica *strain. GO terms from top to bottom display biological process, cellular component, and molecular function domains

Since YLE155 showed both exceptional flask-scale performance and the most differentiated gene expression, GO enrichment analysis focused on this strain. In Fig. 9, the statistically significant enriched GO terms for YLE155 are presented for the three GO domains—biological process (GO:BP), cellular component (GO:CC) and molecular function (GO:MF). The same analysis was performed for YLE111 (Additional file 1: Figure S3). Results show that after osmotic stress imposed from high glycerol concentration, the most enriched GO terms in YLE155 (encompassing more than 150 genes each) were localization and transport processes (GO:BP), membrane, integral membrane and intrinsic membrane components (GO:CC), as well as transporter and transmembrane transporter activity (GO:MF). All the alterations in gene expression, causing the enrichment of the abovementioned GO terms, resulted in the strengthened-adapted phenotype of YLE155, which was evidenced through its growth performance and lipid formation ability.

### Identification of genome sequence perturbations in evolved strains

The assembled contigs for each variant were also compared to those of the parental strain for mismatches and gaps to identify mutations and alterations in the expressed genes’ sequence. In strain YLE111, 235 mismatches and 27 gaps were identified. No mutations were found in the transcript sequences except for the case of YALI0B07623p, coding for a BUD4 domain protein (a 12 amino acid sequence) present in the parental strain, which is spliced in YLE111. Strain YLE155 contained 207 mismatches and 34 gaps. No mutations were found in the transcript sequences. The YALI0F06908p, a probable transport protein, is spliced in and translated correctly in YLE155, whereas it is unspliced in the parental strain. Additionally, YALI0E02178p, with similarity to a transposase, is spliced in YLE155 in the same way as in CLIB122 reference strain, at the 3’ end, unlike the parental. Strain YLE198 contained 173 mismatches and 26 gaps. Mutations were identified in YALI0D11880p (1055F > I, 1057S > A, 1059S > A), another protein with BUD4 homology. Altered splicing was observed for YALI0B04884p with homology to redox sensitive pirin protein, which is spliced in YLE198, whereas in parental cells includes an intron with stop codon.

Strains YLE469 and YLE471 originated from EMS mutagenesis and subsequent evolution, were expected to contain increased number of genetic perturbations. Strain YLE469 contained 123 mismatches and 29 gaps. One gene YALI0F09746p, encoding for a Serine/Threonine Protein Kinase C (homologue of ScYBL105c PKC1), contained a single amino acid deletion of K391. As in strain YLE111, the BUD4 homologous protein YALI0B07623p, was spliced to remove the extra 12 amino acids. Interestingly, in yeasts PKC1 and BUD4 participate in the formation of the septin ring during bud formation. Strain YLE471 harbored 188 mismatches and 33 gaps. Mutations in four genes were identified: 1) YALI0E17105p, a probable phospholipid transporting ATPase 2 (321S > Y); 2) YALI0F31273p uracil phosphoribosyl transferase (88G > D); 3) YALI0F13673p a Guanine nucleotide exchange factor (RhoGEF) for Rho/Rac/Cdc42-like GTPases with a shift in the translation frame at 953aa, likely leading to a shorter protein, which also plays a role in septin ring formation; and 4) YALI0B05940p E3 ubiquitin ligase (471 T > I) homologous to ScTOM1, an enzyme responsible for mRNA export and histone regulation and septin ring regulation. Three genes showed altered splicing: 1) YALI0B13310p, related to yeast ECM14, a fungal protein required for cell wall assembly that contained 6 additional amino acids in YLE471; 2) YALI0D01067p, related to IML1 protein, involved in oxidative stress and amino acid deprivation that contained 4 additional amino acids, identically to the reference strain CLIB122; and 3) YALI0E11165p, an unknown protein which contained 3 aa shorter sequence in a spliced junction.

### Batch fermentation performance of YLE155 on crude glycerol

**Fig. 10:**
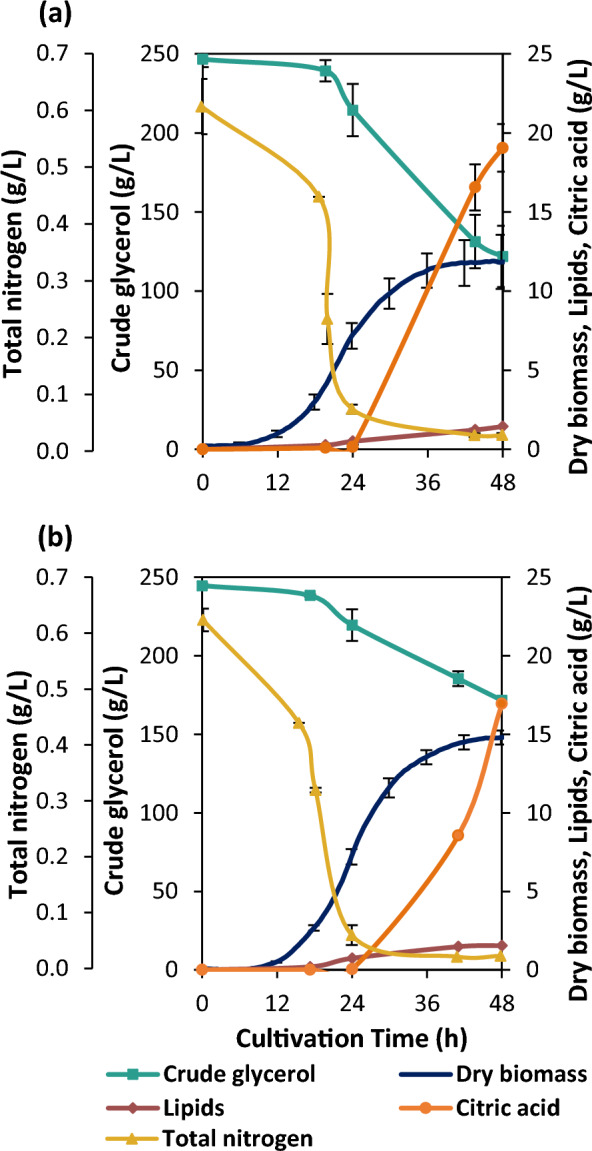
Bioreactor fermentation kinetics of **a** *Y. lipolytica* parental strain and **b** YLE155 evolved strain, grown in synthetic medium, supplemented with 20% v/v crude glycerol under pH 6, 1 vvm, 800 rpm and 30 °C for 48 h. Error bars indicate standard deviation. Number of biological replicates (N = 3)

The enhanced performance of the evolved strain YLE155 was confirmed under well-defined experimental conditions in a lab-scale bioreactor. Bioreactor experiments of the YLE155 evolved *Y. lipolytica* strain in synthetic medium, containing 20% v/v (approx. 245 g/L) biodiesel-derived crude glycerol, showed a significant difference in glycerol consumption compared to the parental. In the first 24 h, YLE155 consumed crude glycerol in a similar manner as the parental. During the next 24 h, it became obvious that glycerol was taken up 2.5 times slower by YLE155, compared to the parental (Fig. 10). Specifically, the maximum uptake rate of glycerol reached approx. 2.8 g/L/h and 5.7 g/L/h for the YLE155 and the parental, respectively. However, at 48 h, 25.4% of the carbon of the consumed glycerol was directed to dry biomass in YLE155, whereas 12.6% was the respective value of WT_par_ (Additional file 1: Table S3). By monitoring the carbon balance, it was evident that the adaptation of YLE155 to stressful glycerol concentration led to a phenotype with lower metabolic demands and altered carbon distribution. By the end of the process, YLE155 reached 14.9 g/L of dry biomass concentration. The obtained physiological data, presented in Fig. 10, underline the main phenotypic alterations caused by the adaptation to high glycerol concentration. Even though the 12-h lag phase was similar in both WT_par_ and YLE155 fermentations, the end of the exponential phase was substantially extended in the second case, resulting in higher biomass and lipid concentration values. Growth rate was significantly increased in YLE155 cultivation, reaching 0.37 ± 0.01 h^−1^, whereas growth rate of the WT_par_ was equal to 0.19 ± 0.01 h^−1^. Lipids were mainly formed during exponential growth phase, which could be attributed to the continuation of cell proliferation. Nitrogen consumption was similar during both fermentations being depleted after the first 24 h. Citric acid was secreted in both fermentations after the nitrogen depletion, however citric acid production rate of YLE155 was 40% lower, secreting in total twofold less citric acid at 42 h compared to the WT_par_. The production metrics of the fermentation products and by-products are presented in Table 2.

**Table 2:** Dry biomass (DB), lipid (L) and citric acid (CA) production metrics derived from bioreactor fermentations

Cultivation time (h)	24							48						
Strain No	Y_DB/Gl_	Y_L/Gl_	Y_L/DB_	Y_CA/Gl_	P_DB_	P_L_	P_CA_	Y_DB/Gl_	Y_L/Gl_	Y_L/DB_	Y_CA/Gl_	P_DB_	P_L_	P_CA_
WT_par_	0.35	0.03	0.07	0.01	0.29	0.02	0.01	0.10	0.01	0.12	0.19	0.26	0.03	0.40
YLE155	0.43	0.04	0.10	0.00	0.30	0.03	0.00	0.21	0.02	0.10	0.23	0.31	0.03	0.35

Cultivation of YLE155 and WT_par_ in 1.75L of synthetic medium, supplemented with 20% v/v crude glycerol. Yields (Y) are calculated as g/g and productivity (P) units are g/L/h after 24 and 48 h

Number of replicates (N = 3). (Standard Deviation < 10%)

The differences in productivities and yields between the two strains are of key importance since the yields of dry biomass and lipids on glycerol were higher in YLE155, almost double after 48 h. Lipid content (Y_L/DB_) slightly increased during exponential growth phase until 24 h and kept a constant value until the late beginning of stationary growth phase (48 h). Gas chromatography analysis, after lipid extraction and transesterification, showed similar lipid profiles in both YLE155 and WT_par_, indicating that TAG composition was not affected, throughout the evolution of YLE155 (Additional file 1: Table S4). Citric acid yield of YLE155 was zero after the first 24 h. However, its secretion rate showed a significantly increasing trend in proportion to cultivation time, by doubling, between 42 and 48 h.

## Discussion

ALE provided valuable information of adaptive alterations *of Y. lipolytica*, using increased glycerol concentration in the medium, in three strategies (increased pure glycerol, increased pure glycerol after EMS mutagenesis, and increased crude glycerol). Phenotypic differences among various isolates, regarding growth and lipid content, showed that biomass and lipid production can happen either synergistically or antagonistically, indicating heterogeneity between strains. It is argued, that trade-off alterations might occur after ALE, when the stressing factor is partially eliminated [46]. Moreover, cell aggregation is a common survival feature in yeasts during adaptation [47]. Therefore, colony isolation and characterization were critical to maneuver possible desired or undesired traits as well as to connect phenotypic changes to genetic events. Differential gene expression analysis for the 5 superior evolved strains (EVS) and the wild type, highlighted common cell functions that adapted in all EVS, independently of their ALE origin, such as nucleosomal structure and regulation of transcription. Interestingly, in a study assessing metabolic changes in *S. cerevisiae* by measuring amino acid biosynthesis in deletion library mutants, it was found that global “indirect” participants such as chromatin regulation and transport pathways were highly enriched for metabolic alterations. Amino acid biosynthesis is responsible for a major fraction of the metabolic flux in exponentially growing cells. When all gene deletions were ranked according to their metabolic impact, the dominant role of histone modifications and its corresponding protein machinery (SWI/SNF, RSC complex, INO80, SAGA complex etc.) became evident. Only intracellular transport had a comparable metabolic impact. A plausible evolutionary explanation for this finding in our data could be that, as metabolism is one of the oldest cellular systems that require regulation, the general gene expression machinery, older than gene-specific transcription factors, would have primarily evolved to regulate metabolism. This is corroborated by the fact that specialized transcriptional systems are mostly dispensable for exponential metabolism [48]. It has been reported that syncytia tend to form during spontaneous mutations for adaptation and growth purposes [49]. Although this was not seen microscopically in our study, we observed a dramatic drop at OD measurements after inoculation (Additional file 1: Figure S4), indicating morphological adaptation shifts taking place to cope with the presence of high glycerol concentration. YLE111 and YLE155, the most differentiated evolved strains, overexpressed genes, known to encode proteins participating in TAG biosynthesis through glycerol catabolism [41]. On scale-up fermentations, significantly lower glycerol consumption rate was evident in YLE155, from 24 to 48 h of cultivation, compared to parental strain. However, YLE155 cultivation resulted in higher production yields. In the case of YLE155, a simplified carbon balance estimation revealed that the adaptation of YLE155 to high glycerol concentrations yielded a strain with lower metabolic demands (lower glycerol uptake rate) and a rerouted carbon flux directing more carbon into biomass [36, 37]. Furthermore, this notable drop of glycerol consumption rate, in parallel with the enhanced growth, can be associated to the altered gene expression profile of YLE155, where biological processes involved in molecule transport are considerably enriched.

Examination of transcript contigs identified changes in exon utilization and mutations in the evolved strains. Unlike *S. cerevisiae*, *Y. lipolytica* is the hemiascomycete with the most intron-rich genome sequenced to date, and has several unusual genes with large introns or alternative transcription start sites, or introns in the 5' UTR. Our results show that when adapting to glycerol stress, the organism utilized alternative splicing to change protein structure and function [50]. Seven proteins are changed by alternative splicing. One of them, a BUD4 homologue is spliced in both YLE111 and YLE469 strains. Only one out of the seven spliced forms, which coded for a pirin protein, contained a stop codon in the intron sequence in the parental strain and is likely degraded by a functional NMD pathway [50]. From the six mutated genes that we identified, four of them (YALI0D11880p a BUD4 homologue; YALI0F09746p, a Protein Kinase C homologue; YALI0F13673p, a Guanine nucleotide exchange factor (RhoGEF); and YALI0B05940p, E3 ubiquitin ligase ScTOM1 homologue) participate in septin ring regulation during the budding process together with BUD4 [51, 52]. Septins are conserved cytoskeletal proteins that bind and hydrolyze GTP. In the case of *S. cerevisiae*, septins form a ring at the bud neck—the constriction between mother and future daughter cell. In the filamentous fungi *Aspergillus nidulans* and *Ashbya gossypii*, a similar event occurs during hyphal branching. The Cdc42 GTPase of the Rho family plays a crucial role in the recruitment of septins to the bud site, and cycles of Cdc42 GTP binding and hydrolysis are required for septin collar formation [53]. Rho1 and Pkc1 are involved in the cell wall integrity pathway, which responds to a variety of cell wall stresses such as osmotic stress and activates a mitogen-activated protein kinase (MAPK) cascade response [52]. The septin architecture is dynamically remodeled at the division site during the cell cycle, and this involves the regulation by post-translational modifications such as phosphorylation, acetylation, ubiquitination and SUMOylation, and septin-associated proteins [54, 55].

## Conclusion

*Yarrowia lipolytica MUCL 28849* wild type strain underwent adaptive laboratory evolution, increasing step-wise the concentration of pure or crude glycerol up to 20% v/v (252.3 g/L). Moreover, random chemical mutagenesis with EMS preceded to a third ALE strategy employing increasing concentration of pure glycerol. Isolates showed major enhancement in dry biomass formation, heterogeneity and in some cases, an increase in lipid concentration. ALE has proven to be an effective approach to create novel strains with improved traits against adverse conditions. Evolved strain transcriptomes, provided a clear framework of the alterations related to principal biological processes that are crucial for adaptation. Global changes in the nucleosome structure and modifications could quickly drive major adaptive changes to high osmotic glycerol stress, which subsequently leads to further differentiation for increasing fitness. Bioreactor fermentations exhibited a more efficient crude glycerol utilization towards biomass formation, compared to the parental strain. The developed evolved strains can be used as suitable “chassis” in rational engineering approaches for increased lipid accumulation and production of chemicals with high-added value utilizing crude glycerol as an inexpensive carbon substrate.

## Materials and methods

### Strain, media and culture conditions

The wild type *Yarrowia lipolytica* MUCL 28849 (WT_par_), used in this study, was obtained from BCCM/MUCL (Agro) Industrial Fungi & Yeasts Collection, Belgium. The WT_par_, the derived evolved populations, and the isolated evolved strains were preserved at -80 °C in 25% v/v pure glycerol and plated on YPG-agar (yeast extract 10 g/L; peptone 20 g/L; glycerol 2% v/v) prior to each broth culture. The first broth precultures were carried out in 5 mL YPG complex medium, whereas the second precultures and the main cultures were performed in synthetic nitrogen-limited media. Composition of the latter was adapted and modified from Egermeier et al. [7], and consisted of (g/L): (NH_4_)_2_SO_4_, 3.0; KH_2_PO_4_, 2.0; Na_2_HPO_4_ × 2 H_2_O, 2.6; MgSO_4_ × 7 H_2_O, 1.0; CaCl_2_ × 2 H_2_O, 0.2; FeCl_3_, 0.02; (mg/L): Thiamin-HCl, 1.0; H_3_BO_3_, 0.5; CuSO_4_ × 5 H_2_O, 0.06; KI, 0.1; MnSO_4_ × H_2_O, 0.45; ZnSO_4_ × 7 H_2_O, 0.71; Na_2_MoO_4_ × 2 H_2_O, 0.23. Pure (≥ 99.5%) and crude (≥ 90–92%) glycerol were used as carbon sources. Crude glycerol was received from a biodiesel production plant (Fytoenergeia/NewEnergy S.A., Greece) and used without any pretreatment. Media were sterilized at 121 °C for 20 min apart from the mineral stock, containing all the micronutrients, which was sterilized through filter sterilization. Glycerol concentration ranged from 2.5 to 20% v/v (31.5 to 252.3 g/L) while C/N ratio from 22.6 to 181.

Fermentations were performed either in 96-well microtiter plates for screening experiments (0.2 mL) or in 100-mL flasks for strain characterization (25 mL, 150 rpm, 30 °C) using synthetic nitrogen-limited media. A 3-L bench-top bioreactor (BioFlo120, Eppendorf, Germany) was also used to run batch cultivations, to evaluate the performance of the selected evolved strain at high crude glycerol concentration (20% v/v).

### Analytical methods

Cell growth was monitored using OD_600nm_ (BioPhotometer, Eppendorf, Germany), whereas dry biomass (DB) was determined as follows. Aliquots of 1 mL were centrifuged (3750 g × 6.5 min—Heraeus Megafuge 16R, Thermo Fisher Scientific Inc., MA, USA) and the obtained cell pellets were washed twice prior to drying at 60 °C until reaching a constant weight. After the first centrifugation, the supernatant of the fermented broth was diluted, filtered (0.45 μm) and used for the detection of the residual glycerol and secreted citric acid using high performance liquid chromatography (HPLC). For the HPLC system (CBM-20A, Shimadzu Europa GmbH, Germany), a Shodex Sugar SH1011 (8.0 mm I.D. × 300 mm) column was coupled to a Refractive Index Detector (RID-10A, Shimadzu Europa GmbH, Germany) and a UV detector (SPD-M20A, Shimadzu Europa GmbH, Germany). 14 mM sulfuric acid, at a flow rate of 0.4 mL/min, was used as the mobile phase. Residual nitrogen concentration was quantified using a total nitrogen analyzer (TNM-L, Shimadzu Europa GmbH, Germany).

To extract the intracellular lipids of *Y. lipolytica*, cell pellets were collected, after removing the consumed substrate through centrifugation (3750 g × 6.5 min), washed twice with dH_2_0 and stored at − 20 °C. The frozen pellets were lyophilized to a constant weight, and 0.1 g of freeze-dried biomass was diluted in 10 mL of methanol/hexane (3:5, v/v) [56]. After three 5-min cycles of ultra-sonication (Cole-Parmer Ultrasonic cleaner, Cole-Parmer Instrument Company, Vernon Hills, IL, USA), samples were vortexed for 1 h to disrupt cell membrane [57]. For phase separation, 1 mL of saline solution (0.9% w/v, NaCl) was added and the two-phase sample was shortly vortexed before centrifugation. The non-polar layer was withdrawn and purified. The remaining solvent (hexane) was evaporated until constant lipid mass. Lipids were resuspended in hexane either to be stored at − 20 °C or to perform transesterification. Lipids were transesterified according to Tai et al. [57]. Fatty acid methyl ester (FAME) composition was identified through gas chromatography (GC-FID) analysis (GCMS-QP2010, Shimadzu Europa GmbH, Duisburg, Germany), equipped with a capillary column SP-2340 (60 m × 0.25 mm, 0.20 μm film thickness) (Supelco, PA, USA). A commercial FAME mix (Supelco 37 Component FAME Mix, Sigma-Aldrich, St. Louis, MO, USA) was used for comparison of the relative percentage of each fatty acid, determined by internal normalization of the chromatographic peak area.

A colorimetric method was developed, based on sulfo-phospho-vanillin (SPV) reaction [58, 59], to determine lipid production during both flask and bioreactor fermentations. In a glass tube, 100 μL of culture broth (diluted with dH_2_O depending on cell density) and 2 mL of dense H_2_SO_4_ were mixed and incubated in a thermoreactor (Spectroquant Thermoreactor TR 620, Merck KGaA, Germany) at 100 °C for 10 min. After a 5-min rest in a water bath at room temperature (RT), 5 mL of phospho-vanillin solution (65% v/v H_3_PO_4_/H_2_O, 0.12 g/L vanillin) were added. Samples were incubated for 15 min at 37 °C prior to a 10 min rest in a RT water bath. Absorbance (corresponding to mg of lipids) was measured at 530 nm (Spectroquant® UV/VIS Spectrophotometer, Pharo 300, Merck KGaA, Germany). Commercial sunflower oil was used for the construction of the calibration curve (0 – 0.135 mg).

Flow cytometry (Attune™ NxT Acoustic Focusing Cytomerer, Invitrogen ThermoFisher Scientific, MA, USA) was also used to quantitate intracellular lipids. Lipids were stained with bodipy dye (493/503 nm) by adding of 10 μL of bodipy (10 μg/mL DMSO) and incubating for 30 min at 30 °C. The cells were washed twice with phosphate buffered saline solution (× 10) before flow cytometry analysis. Both stained and unstained parental strain cells were used as control samples. Morphology and lipid droplets were observed through fluorescent microscopy (ZOE™ Fluorescent Cell Imager, Bio-Rad Laboratories Inc., Hercules, CA, USA).

### Adaptive Laboratory Evolution (ALE)

In this study, ALE was applied to *Y. lipolytica* using as stressing factor the gradually increasing concentration of glycerol. Both pure and crude glycerol were used; 20% v/v was the highest concentration tested. The osmolality of the synthetic medium was determined for each pure and crude glycerol concentration using a freezing point osmometer (OSMOMAT 3000, Gonotec GmbH, Germany). In addition, random chemical mutagenesis with EMS was employed to enhance the natural mutation rate (30 μL EMS were added in 1 mL YPG culture of OD_600nm_ 6 and incubated for 15 min at 30 °C). In total, three different strategies were implemented (Fig. 1). The starting populations were the wild type *Y. lipolytica*, grown in increasing pure and crude glycerol concentration, and the EMS_mut_ grown in increasing pure glycerol concentration. Three 100-mL flasks of 25-mL synthetic medium, two supplemented with 9% v/v pure glycerol and one with 9% v/v crude glycerol, were inoculated at an initial OD_600nm_ = 0.23 ± 0.05. Within exponential growth (24 h), a number of cells from each of the three flasks was inoculated into fresh medium, generating the first subculture of the ALE experiment. Serial subcultures were employed every 24 h, while the initial OD was adjusted at the same value of 0.23 ± 0.05. The ΔOD/Δt ratio was monitored and when a stable phenotype was achieved, the concentration of glycerol, as well as the C/N ratio were elevated. Glycerol concentration reached 20% v/v in both pure and crude glycerol experiments after a total of 104 passages. The three populations WT_ev.pure_, WT_ev.crude_ and EMS_ev.pure_ were cryo-preserved in 25% v/v pure glycerol at − 80 °C after every few rounds.

### Selection and characterization of superior evolved strains

Evolved populations were cultivated for 24 h in 15% v/v pure and crude glycerol synthetic medium, and then dilutions were streaked in synthetic medium agar plates, containing 18% v/v pure and crude glycerol, to isolate single colonies. Microtiter plate growth assays of EVS were conducted in 96-well plates to identify those with the highest biomass and lipid yields. 15% v/v pure glycerol was added in the medium and OD_600nm_ was recorded for 48 h at 30 °C and semi-continuous shaking at 120 rpm (6 min/1 h) (Microtiter plate reader Spark, Tecan, Switzerland). The plates were covered with a gas permeable sealing membrane (Breathe-Easy®, Diversified Biotech Inc., MA, USA) to ensure aeration. The best performing evolved strains were assessed for lipid production (pre-final selection). Final selection tests were carried out comparing the evolved strain performance when growing in 25 mL of synthetic medium, containing 15% v/v pure glycerol versus 15% v/v crude glycerol. Growth, dry biomass and lipid content were monitored for 72 h and their fold-change compared to the parental strain was estimated. In all cases, the parental strain was used as control. The statistical significance between the performance of each EVS and the WT_par_ was estimated using one-way ANOVA and post hoc tests. The p-value was equal to 0.05 for one-way ANOVA and the statistical threshold for the post hoc tests was estimated according to the Bonferroni correction. Statistical analysis was performed using Microsoft Excel® software v.15.0.

### RNA sequencing analysis and Investigation of differentially expressed genes

Differential gene expression analysis between WT_par_ and superior EVS was performed through NGS. RNA extraction was conducted by firstly growing each strain in high pure glycerol substrate for 24 h (synthetic medium with 15% v/v pure glycerol, working volume, WV = 20 mL, T = 30 °C, 150 rpm) and in 3 biological replicates. The required biomass was collected in pellet through centrifugation (3750 g × 6.5 min, IEC FL40 Centrifuge, Thermo Fisher Scientific, MA, USA). For RNA extraction, a modified version of the protocol of Invitrogen Trizol User Guide was used. After pellet resuspension in Trizol reagent (Invitrogen, CA, USA) and homogenization of the lysate, 0.4 g acid washed glass beads (450–550 μm mean diameter) were added and samples were disrupted and homogenized in a Retsch MM300 TissueLyser for 5 min at 30 Hz (Verder International BV, Netherlands). Extraction with chloroform was performed twice (0.2 at first and then 0.4 mL). RNA concentration was measured on a Qubit 4.0 Fluorometer using the Qubit® RNA BR assay kit (Invitrogen, CA, USA). mRNA was enriched with NEBNext Poly(A) mRNA Magnetic Isolation Module (NEB #E7490) using oligo d(T) beads for binding the poly(A) tail of eukaryotic mRNA and libraries were constructed using Illumina’s NEBNext® Ultra™ II RNA Library Prep Kit for Illumina® (NEB #E7770) (New England Biolabs, MA, USA). PCR products and libraries were purified to remove unincorporated primers and primer-dimer species using Agencourt AMPure XP magnetic beads (Beckman Coulter—Life Sciences, IN, USA). All libraries were quantified with fluorometric quantification using the Qubit® dsDNA BR assay kit (Invitrogen, CA, USA), and their size and quality were evaluated on a 5200 Fragment Analyzer system (Agilent Technologies Inc., CA, USA) using the DNF-474–0500 kit. The molarity of libraries was assessed by a qPCR conducted on a Rotor-Gene Q thermocycler (Qiagen, Germany) with the KAPA Library Quantification kit for Illumina sequencing platforms (KAPA BIOSYSTEMS, MA, USA). Libraries were sequenced on a NextSeq 500 platform using the NextSeq 500/550 High Output Kit v2.5 (75 Cycles) (Illumina Inc., CA, USA).

Raw reads were trimmed and quality filtered using TrimGalore (v0.6.7). After trimming, adapter sequences, ambiguous bases (N) and reads with quality score below 30 were removed from the data. Differential expression analysis was performed according to the “new Tuxedo” pipeline [60]. Specifically, filtered reads were aligned against the *Yarrowia lipolytica* reference genome (GCF_000002525.2) using HISAT2 (v2.1.0). Transcript assembly and quantification were performed with StringTie (v2.0.6). Output data were imported into R (v4.2.1) and differential expression analysis was conducted using the Ballgown package (v2.28.0). For gene annotation, the assembled contigs were aligned against the *Yarrowia lipolytica* reference proteome (UP000001300), downloaded from UniProt [61], with e-value threshold 0.001 using DIAMOND (v2.0.1) [62]. As differentially expressed genes (DEGs) were considered those with p-value lower that 0.001 and absolute log2(FC) higher than 1. For GO functional enrichment analysis, the gprofiler2 package (v0.2.1) was employed [63]. The statistical significance threshold was set to p-value < 0.05 and was computed using the g:SCS multiple testing correction method. Data visualization was performed using the ggplot2 (v3.3.6) [64], pheatmap (v1.0.12) (Additional file 1: Figure S5) [65], UniprotR (v2.2.0) [66] and enrichplot (v1.16.1) [67] packages.

### Identification of genome sequence perturbations in evolved strains

For the detection of genetic perturbations in the expressed sequences, from all *Y. lipolytica* strains, the data of the three biological replicates of each strain were merged after trimming. To generate expressed contigs, reference genome guided assembly was performed with Trinity (v2.8.5) [68] using the *Y. lipolytica* CLIB122 (assembly ASM252v1) genome as reference, following the pipeline outlined at the Canadian bioinformatics workshop for genome assembly of all strains RNA-Seq [69]. Data for the assembly were prepared by firstly converting the GFF file of the reference genome to GTF using gffread [70], followed by the extraction of splicing sites and exon records with HISAT2 (v2.1.0) [71]. HISAT2 was also used for the alignment of the wild type *Y. lipolytica* MUCL 28849 to the reference. To validate the quality of the assembled transcriptomes, BUSCO tool (v5.3.0) was employed [72]. Variant calling was performed using the pipeline developed at [73]. More specifically, the BWA-MEM algorithm (v0.7.17-r1188) was employed to align the reads from the EVS to the assembled reference transcriptome. Sorting of aligned reads, by coordinates and statistics was performed with SAMtools (v1.14) [74]. To count the read coverage for variant calling and detect the single nucleotide variants (SNVs), we used BCFtools (v1.9) with the “mpileup” and “call” commands [75]. The variants were filtered using the BCFtools “filter” option. To identify the contigs of the parental strain, command line blast was used [76]. A database was built for the reference genome of *Y. lipolytica* with the command “makeblastdb” and the wild type and each mutant strain were aligned to the database using blastn. This process was repeated, building a database for the wild type strain and aligning each mutant strain to it. Subsequently, we examined manually the aligned transcripts of each strain for mismatches and gaps.

### Bioreactor fermentations

Batch fermentations of the resulting superior EVS and the wild type *Y. lipolytica* were performed using a 3-L bench top bioreactor (BioFlo 120, Eppendorf, Germany) in order to distinguish kinetic differences, involving biomass and lipid production as well as glycerol uptake, while scaling up. Critical process metrics were evaluated i.e., growth rate, biomass formation, lipid accumulation, glycerol and nitrogen uptake rate as well as citric acid secretion rate under controlled fermentation conditions (pH 6, aeration rate 1 vvm and agitation speed 800 rpm). The WV was set at 1.75 L and the yeast was grown in synthetic medium, supplemented with 20% v/v of crude glycerol, for 48 h at 30 °C. The reactor was equipped with a pH sensor (405-DPAS-SC, © METTLER TOLEDO, Switzerland) and a dissolved oxygen probe (DO, InPro6860i, © METTLER TOLEDO, Switzerland). Real-time monitoring of dry biomass was possible using a submerged optical density probe (OD, Dencytee Unit, Hamilton Bonaduz AG, Switzerland) and correlating the OD values (R^2^ > 0.999) (Additional file 1: Figure S6). The pH was regulated by automated addition of 5 N H_2_SO_4_ and 5 N NaOH. To inoculate the bioreactor, three serial precultures were prepared, with the third culture, incubated in a 500 mL-flask (WV: 100 mL). The preculture was centrifuged and the supernatant was removed. The pellet was suspended using 5 mL of sterile deionized water prior to inoculation to keep the WV of the bioreactor constant.

## Supplementary Information

**Additional file 1: Table S1.** Osmolality of synthetic medium (SM), supplemented with different concentrations of pure and crude glycerol. **Figure S1**. Monitoring of cell morphology during the cultivation of Yarrowia lipolytica MUCL 28,849 wild type (WT_par_) and the evolved strain YLE469, in 25 mL synthetic medium, containing 15% v/v pure glycerol. Letters indicate cultivation timepoints, **(a)** 24 h, **(b)** 48 h, **(c)** 72 h and **(d)** 96 h. YLE469 strain was obtained after EMS mutagenesis and adaptive laboratory evolution through 520 generations of gradual increase of pure glycerol into the medium (9–20% v/v). Snow-flake morphology was observed in two biological duplicates. **Figure S2**. Cell morphology of the evolved strain YLE469, cultivated in 25 mL synthetic medium, containing 15% v/v pure glycerol, for 48 h. YLE469 strain was obtained after EMS mutagenesis and adaptive laboratory evolution through 520 generations of gradual increase of pure glycerol into the medium (9–20% v/v). Snow-flake morphology was observed in two biological duplicates. **Table S2.** Number of reads obtained after sequencing and quality filtering of Yarrowia lipolytica 28,849 and evolved strains (after ALE). For each strain, the total number of sequences obtained for the three biological replicates is presented. **Figure S3.** Dotplot of gene ontology (GO) enrichment analysis for YLE111 evolved Yarrowia lipolytica strain. GO terms from top to bottom display biological process, cellular component, and molecular function domains. **Figure S4.** %OD drop of Yarrowia lipolytica cells during lag growth phase, grown in 50 mL synthetic medium containing different crude glycerol concentrations (5, 7.5, 10, 15, 20% v/v) using as inoculum (a) preculture from YPG broth (b) preculture cultivated first in YPG broth and then in synthetic medium for preadaptation. The drop was observed immediately after the inoculation and its duration increased with increasing glycerol concentration. **Figure S5.** Heatmap showing the expression levels of all genes in the strains WT_par_, YLE111, YLE155, YLE198, YLE469 and YLE471. Gene expression levels were normalized across all samples using z-score. Blue and red color indicates high and low expression levels, respectively. Sample (columns) and gene (rows) clustering was performed based on the respective euclidean distances. **Figure S6.** Correlation between dry biomass (g/L) and optical density (OD). Dry biomass was collected from a bioreactor fermentation at different cultivation time points, while OD values were obtained from a submerged OD sensor. **Table S3.** Estimated carbon balance of WT_par_ and YLE155, cultivated in 1.75 L synthetic medium, supplemented with 20% v/v crude glycerol for 48 h. Fermentations were conducted in triplicates (N = 3). Carbon (mol) of glycerol and citric acid was estimated based on their chemical formula and calculated mass. According to Celińska et al., the percentage of carbon in dry biomass was equal to 50% (g/g) [77]. **Table S4.** Fatty acid (FA) composition of the intracellular lipids of WT_par_ and YLE155, cultivated in 1.75 L synthetic medium, supplemented with 20% v/v crude glycerol for 48 h. Fermentations were conducted in triplicates (N = 3).

## Data Availability

All data generated or analyzed during this study are included in this published article, its supplementary information files and at NCBI SRA repository, BioProjectID: PRJNA922115.

## Abbreviations

*Y. lipolytica*: *Yarrowia lipolytica*
ALE: Adaptive Laboratory Evolution
EMS: Ethyl Methanesulfonate
WT_par_: *Yarrowia* *lipolytica* MUCL 28849 parental stain
EMS_mut_: *Yarrowia* *lipolytica* after EMS mutagenesis
EVS: Evolved Strains
TAG: Triacylglycerol
YLE(number of isolate): Individual evolved strain coding
DEGs: Differentially Expressed Genes
GO: Gene Ontology
BP: Biological Process
CC: Cellular Component
MF: Molecular Function
DB: Dry Biomass
L: Lipids
CA: Citric Acid
Y_x/y_: Yield (gx/gy)
P_x_: Productivity (gx/L/h)
