# Supplementary material for: Novel evolved Yarrowia lipolytica strains for enhanced growth and lipid content under high concentrations of crude glycerol
Source: Microb Cell Fact. 2023 Mar 31;22:62. doi: 10.1186/s12934-023-02072-8 (PMC10067222; doi:10.1186/s12934-023-02072-8)
Supplement: Supplementary file 1 — Additional file 1: Table S1. Osmolality of synthetic medium (SM), supplemented with different concentrations of pure and crude glycerol. Figure S1. Monitoring of cell morphology during the cultivation of Yarrowia lipolytica MUCL 28,849 wild type (WTpar) and the evolved strain YLE469, in 25 mL synthetic medium, containing 15% v/v pure glycerol. Letters indicate cultivation timepoints, (a) 24 h, (b) 48 h, (c) 72 h and (d) 96 h. YLE469 strain was obtained after EMS mutagenesis and adaptive laboratory evolution through 520 generations of gradual increase of pure glycerol into the medium (9–20% v/v). Snow-flake morphology was observed in two biological duplicates. Figure S2. Cell morphology of the evolved strain YLE469, cultivated in 25 mL synthetic medium, containing 15% v/v pure glycerol, for 48 h. YLE469 strain was obtained after EMS mutagenesis and adaptive laboratory evolution through 520 generations of gradual increase of pure glycerol into the medium (9–20% v/v). Snow-flake morphology was observed in two biological duplicates. Table S2. Number of reads obtained after sequencing and quality filtering of Yarrowia lipolytica 28,849 and evolved strains (after ALE). For each strain, the total number of sequences obtained for the three biological replicates is presented. Figure S3. Dotplot of gene ontology (GO) enrichment analysis for YLE111 evolved Yarrowia lipolytica strain. GO terms from top to bottom display biological process, cellular component, and molecular function domains. Figure S4. %OD drop of Yarrowia lipolytica cells during lag growth phase, grown in 50 mL synthetic medium containing different crude glycerol concentrations (5, 7.5, 10, 15, 20% v/v) using as inoculum (a) preculture from YPG broth (b) preculture cultivated first in YPG broth and then in synthetic medium for preadaptation. The drop was observed immediately after the inoculation and its duration increased with increasing glycerol concentration. Figure S5. Heatmap showing the expression levels [file 12934_2023_2072_MOESM1_ESM.docx]

**Supplementary material**

***Table S1.*** Osmolality of the synthetic medium, supplemented with different concentrations of pure and crude glycerol.

| Glycerol (% v/v) | Osmolality_Pure Glycerol_ (mOsmol/kg) | Osmolality_Crude Glycerol_ (mOsmol/kg) |
| --- | --- | --- |
| 9 | 1,682 ± 0 | 2,241 ± 2 |
| 12 | 2,334 ± 2 | 3,273 ± 23 |
| 15 | 2,607 ± 25 | 3,733 ± 9 |
| 20 | 3,793 ± 37 | 5,619 ± 16 |

***Figure S1.*** Monitoring of cell morphology during the cultivation of *Yarrowia lipolytica* MUCL 28849 wild type (WT_par_) and the evolved strain YLE469, in 25 mL synthetic medium, containing 15% v/v pure glycerol. Letters indicate cultivation timepoints, **(a)** 24 h, **(b)** 48 h, **(c)** 72 h and **(d)** 96 h. YLE469 strain was obtained after EMS mutagenesis and adaptive laboratory evolution through 520 generations of gradual increase of pure glycerol into the medium (9-20% v/v). Snow-flake morphology was observed in two biological duplicates (Scale bar=25 μm).

***Figure S2.*** Cell morphology of the evolved strain YLE469, cultivated in 25 mL synthetic medium, containing 15% v/v pure glycerol, for 48 h. YLE469 strain was obtained after EMS mutagenesis and adaptive laboratory evolution through 520 generations of gradual increase of pure glycerol into the medium (9-20% v/v). Snow-flake morphology was observed in two biological duplicates (Scale bar=80 μm).

***Table S2.*** Number of reads obtained after sequencing and quality filtering of *Yarrowia lipolytica* 28849 and evolved strains (after ALE). For each strain, the total number of sequences obtained for the three biological replicates is presented.

| A/A | Strain | Raw reads | | Filtered reads | |
| --- | --- | --- | --- | --- | --- |
| 1 | Yl28849_WT | 47,306,349 | 45,754,332 | |  |
| 2 | YLE111 | 39,768,649 | 38,726,175 | |  |
| 3 | YLE155 | 35,851,761 | 35,057,107 | |  |
| 4 | YLE198 | 41,708,058 | 40,401,104 | |  |
| 5 | YLE469 | 36,149,107 | 35,240,551 | |  |
| 6 | YLE471 | 36,364,086 | 35,447,045 | |  |

***Figure S3.*** Dotplot of gene ontology (GO) enrichment analysis for YLE111 evolved *Yarrowia lipolytica* strain. GO terms from top to bottom display biological process, cellular component, and molecular function domains.

***Figure S4.*** %OD drop of *Yarrowia lipolytica* cells during lag growth phase, grown in 50mL synthetic medium containing different crude glycerol concentrations (5, 7.5, 10, 15, 20% v/v) using as inoculum **(a)** preculture from YPG broth **(b)** preculture cultivated first in YPG broth and then in synthetic medium for preadaptation. The drop was observed immediately after the inoculation and its duration increased with increasing glycerol concentration.

***Figure S5.*** Heatmap showing the expression levels of all genes in the strains WT_par_, YLE111, YLE155, YLE198, YLE469 and YLE471. Gene expression levels were normalized across all samples using z-score. Blue and red color indicates high and low expression levels, respectively. Sample (columns) and gene (rows) clustering was performed based on the respective euclidean distances.

***Figure S6.*** Correlation between dry biomass (g/L) and optical density (OD). Dry biomass was collected from a bioreactor fermentation at different cultivation time points, while OD values were obtained from a submerged OD sensor.

***Table S3.*** Estimated carbon balance of WT_par_ and YLE155, cultivated in 1.75 L synthetic medium, supplemented with 20% v/v crude glycerol for 48 h. Fermentations were conducted in triplicates (N = 3). Carbon (mol) of glycerol and citric acid was estimated based on their chemical formula and calculated mass. According to Celińska et al., the percentage of carbon in dry biomass was equal to 50% (g/g) [77].

| Strain No. | WT_par_ | | YLE155 | |
| --- | --- | --- | --- | --- |
| Cultivation time (h) | 24 | 48 | 24 | 48 |
| mol_Consumed Glycerol_ | 1.0 | 4.1 | 0.9 | 2.4 |
| mol_Dry Biomass_ | 0.3 | 0.5 | 0.3 | 0.6 |
| mol_Citric Acid_ | 0.0 | 0.6 | 0.0 | 0.5 |
| mol_Carbon Balance_ | 0.8 | 3.0 | 0.6 | 1.3 |

***Table S4.*** Fatty acid (FA) composition of the intracellular lipids of WT_par_ and YLE155, cultivated in 1.75 L synthetic medium, supplemented with 20% v/v crude glycerol for 48 h. Fermentations were conducted in triplicates (N = 3).

| **Strain No.** | **WT_par_** | | **YLE155** | |
| --- | --- | --- | --- | --- |
| **Cultivation Time (h)** | **24** | **48** | **24** | **48** |
| **Palmitic C16:0** | 18.09 ± 0.02 % | 15.23 ± 0.01 % | 21.69 ± 0.00 % | 18.84 ± 0.02 % |
| **Palmitoleic C16:1 (cis-9)** | 8.57 ± 0.01 % | 8.40 ± 0.02 % | 11.04 ± 0.01 % | 13.34 ± 0.02 % |
| **Stearic C18:0** | 11.90 ± 0.02 % | 9.57 ± 0.01 % | 13.76 ± 0.01 % | 9.49 ± 0.03 % |
| **Oleic C18:1 (cis-9)** | 30.05 ± 0.02 % | 41.71 ± 0.01 % | 31.23 ± 0.01 % | 43.79 ± 0.03 % |
| **Linoleic C18:2 (cis-9,12)** | 31.39 ± 0.04 % | 24.99 ± 0.04 % | 22.28 ± 0.02 % | 14.54 ± 0.01 % |
